# Refractive Outcomes After Cataract Surgery—The Impact of Preoperative Visual Acuity, the Intraocular Lens Model, and the Surgeon’s Experience: An Empirical Analysis of Hungarian and Kosovan Patients

**DOI:** 10.3390/jcm13237013

**Published:** 2024-11-21

**Authors:** Flaka Shoshi, Fitore Shoshi, Agim Xhafa, Zoltán Zsolt Nagy

**Affiliations:** 1 Department of Ophthalmology, Semmelweis University, 1085 Budapest, Hungary; nagy.zoltan_zsolt@med.semmelweis-univ.hu; 2 Department of Ophthalmology, University Clinical Center of Kosovo, 10000 Prishtina, Kosovo; shoshifitore@gmail.com (F.S.); agimx50@gmail.com (A.X.)

**Keywords:** cataract surgery, refractive outcomes, monofocal IOLs, multifocal IOLs, toric IOLs, surgeon’s experience

## Abstract

**Background/Objectives:** Phacoemulsification and intraocular lens (IOL) implantation comprise a standard procedure for cataract treatment. However, minimal refractive error remains a determinant of postoperative results. Our study aimed to evaluate the refractive outcomes and the impact of the surgeon’s experience and the IOL model on Kosovan and Hungarian patients after cataract surgery. **Methods:** This study included the preoperative and postoperative data of 1417 patients scheduled to undergo cataract surgery with IOL implantation at two centers: the Ophthalmology Department of Semmelweis University, Budapest, Hungary, and the Ophthalmology Department of the University Clinical Center of Kosovo, Prishtina, Kosovo. STATA and SPSS were used for statistical analysis. **Results:** The data of 1001 Hungarian and 416 Kosovan patients were included in this study. There was a statistically significant difference between the groups in the 1-month postoperative best-corrected distance visual acuity (BCDVA) (*p* = 0.001); in the Hungarian patients, the 1-month BCDVA was 85.2%, while in the Kosovan patients, it was 49.6%. Of the 14 different IOLs implanted in the Hungarian patients, the AcrySof IQ toric SN6AT, FineVision HP (POD F GF), and 677MTY IOLs resulted in a statistically significant positive impact on the 1-month postoperative visual acuity (*p* < 0.05). The AcrySof SA60AT and Akreos ADAPT AO, implanted in the Kosovan patients, had a statistically significant positive impact on the 1-month postoperative visual acuity (*p* < 0.05). More extensive surgeon experience had a statistically significant positive impact on postoperative outcomes (*p* < 0.00). **Conclusions:** Multifocal and toric IOLs showed superiority in terms of postoperative outcomes in our study; therefore, we conclude that greater surgeon experience, the availability of premium IOLs, and appropriate IOL selection have a considerable impact on refractive outcomes after cataract surgery.

## 1. Introduction

Despite the increased prevalence of age-related cataracts due to the acceleration of the aging process [1], especially in low- and middle-income economies [2], they are considered a preventable cause of blindness. Treatment with phacoemulsification and intraocular lens (IOL) implantation is a standard approach, especially in developed countries. With advances in the IOL power calculation formulas and the ongoing perfection of surgical techniques, cataract surgery is considered a procedure that will ensure high-quality postoperative vision for patients [3,4]. The results from the 2004 Survey of Health, Ageing, and Retirement in Europe (SHARE) indicate that persons with visual impairment have a lower quality of life, reading and concentration problems, and difficulty completing other daily tasks, in addition to feeling irritable, fatigued, and sad [5]. The satisfactory outcomes of the surgery are based, not only on increased visual acuity, but also on a better quality of life [6] and independence from eyeglasses [7] or other refractive correction means. Minimal postoperative refractive error is important with all types of IOLs, but it is crucial when premium IOLs, such as multifocal or toric IOLs, are implanted [8], as they are considered the best solution for patients who want to become independent from eyeglasses after cataract surgery [9,10,11,12]. The postoperative refraction target is between 0.0 and 0.5 diopter (D). In general, it is expected that 90% of patients have a spherical equivalent (SE) refraction within ±1.00 D of the target refraction [13,14]. These postoperative outcomes are sometimes difficult to achieve when routine cataract surgery is performed; specifically, study results suggest that this postoperative refraction is achieved in 75–90% of cases after routine cataract surgery [6,15,16,17].

To achieve these goals, precise preoperative assessment, IOL selection and availability, intraoperative complications, surgeon experience, and postoperative follow-up have a major influence. Precise preoperative IOL planning depends on the axial length (AL), corneal power (K), and anterior chamber depth (ACD); a 1 mm error in the AL results in up to a 3 diopter (D) refractive error, and a 1 D error in the corneal power alters the IOL power for 1 D [18]. Despite the refinement of the IOL power calculation formulas, most large refractive errors greater than 2 D occur due to inaccurate preoperative measurements [13]. Artificial intelligence (AI) has played a major role in the diagnosis, management, and treatment of cataracts, especially in biometry and IOL power calculation formulas [19]. AI-generated IOL power calculation formulas have proven to be very promising in predicting postoperative refraction [20]. Studies have found that, of the AI formulas, the Kane formula, which focuses on improving the accuracy at the extremes of the various ocular dimensions, such as AL, K, and ACD, obtains the most accurate results, and its mean absolute error (MAE) was the lowest among the other AI-generated IOL power calculation formulas [21,22,23,24,25].

Despite the advances in the power calculation formulas, the IOLs, as artificial lenses, have no accommodation ability; therefore, most patients receiving a monofocal IOL require postoperative refractive correction to conduct their daily activities [26]. The results of the ASCRS 2016 Young Eye Surgeons survey indicated that there was an increase in the use of toric IOLs among young ophthalmologists because of their positive refractive outcomes [27]. Therefore, despite the ongoing research in this area, our research group studied these two populations for the first time and compared the postoperative outcomes between the patients treated by a surgeon with 24 years of experience and the patients treated by a surgeon with 11 years of experience in phacoemulsification. Moreover, the latter practiced in a center with no available premium IOLs.

The purpose of our study was to evaluate the refractive outcomes and the impact of the IOL model and surgeon experience on postoperative visual acuity in Kosovan and Hungarian patients after cataract surgery and IOL implantation.

## 2. Materials and Methods

This was a cross-sectional observational multicenter study that included the data of patients scheduled to undergo cataract surgery with IOL implantation at two centers: the Ophthalmology Department of Semmelweis University, Budapest, Hungary, and the Ophthalmology Department of the University Clinical Center of Kosovo, Prishtina, Kosovo. This was a single-surgeon study per center, where all the patients were surgically treated and followed up by the same surgeon in each center. Prof. Dr. Zoltan Zsolt Nagy treated the Hungarian patients, while Dr. Agim Xhafa treated the Kosovan patients. We compared the patients from 2 countries with different levels of development in terms of IOL model availability and phacoemulsification experience to evaluate the impact of these factors on the postoperative outcomes. Prof. Nagy is a surgeon with 24 years of experience, whereas Dr. Xhafa is a surgeon with 11 years of experience in phacoemulsification. All the preoperative measurements were performed using the Lenstar 900 (Haag-Streit, Köniz, Switzerland) biometer. To evaluate the postoperative refractive outcomes, we analyzed 14 different IOL models in the Hungarian patients and 2 IOL models in the Kosovan patients.

### 2.1. Patient Selection and Data Collection

In total, 1417 cataractous patients were included in the study: 1001 patients from Hungary and 416 patients from Kosovo. The data collection process included the following variables: the patient age, the operated eye (right or left), the lens thickness, the uncorrected preoperative distance visual acuity (UDVA), the best-corrected preoperative distance visual acuity (BCDVA), the 1-month postoperative uncorrected distance visual acuity, the 1-month postoperative best-corrected distance visual acuity (BCDVA), and the IOL model.

The data for each patient were compiled using the patient data management system in each center. All the variables were collected from the patients treated by one surgeon in each center, Dr. Nagy in Hungary with 24 years of experience, and Dr. Xhafa in Kosovo with 11 years. This study only included patients who were diagnosed with cataracts as their only ocular disorder. All the patients who were scheduled to undergo cataract surgery but had other ocular comorbidities, such as corneal disorders or previous corneal surgical procedures, were excluded. Those with glaucoma, amblyopia, or posterior segment disorders, such as diabetic retinopathy, age-related macular degeneration (AMD), macular degeneration, or optic neuropathy, were excluded from the study. Only the patients without any preoperative, intraoperative, or up to 1-month postoperative complications and with a standard deviation < 0.2 mm in the AL measurement for the IOL power calculation were considered. Because we aimed to evaluate the postoperative outcomes based on three factors—preoperative visual acuity, the IOL model, and the surgeon’s experience—we excluded all the cases with other comorbidities that could act as confounding factors. To prevent any possible infection, intracameral cefuroxime 1.0 mg/0.1 mL was administered to all the patients following the cataract surgery. Thus, no cases of postoperative endophthalmitis were reported in our study. The data extraction and insertion in our database was completed by the same people in each center to avoid mistakes and mismatches in reporting.

The visual acuity was registered using the decimal system or percentage—hand movement—of 1.0 (0–100%).

This study was conducted in accordance with the Declaration of Helsinki and was approved by the Ethics Committee of the Kosovo Chamber of Doctors (No. 49/2022; date 12 April 2022) and by the Regional Institutional Scientific and Research Ethics Committee at Semmelweis University (SE RKEB 82/2024; Date 14 May 2024).

### 2.2. Statistical Analysis

Quantitative methods were used to analyze the data. This study included patients from two countries—Hungary (*n* = 1001) and Kosovo (*n* = 416).

Descriptive statistics were used to summarize the central tendencies and variability. To analyze the correlation between the variables, we used Pearson’s index of correlation, whereas, to measure the impact of the independent variables on the dependent variable, we used multiple linear regression analysis. For the assessment of the group differences, we used a one-way ANOVA. Three levels of statistical significance, 1% (*p* < 0.01), 5% (*p* < 0.05), and 10% (*p* < 0.1), were used to present the statistically significant differences; this enabled us to highlight not only the most robust results (at 1%) but also those that were moderately strong (at 5%), and suggestive trends (at 10%). This layered approach helped distinguish between varying degrees of evidence, making the results easier to interpret and offering a comprehensive view of the statistical significance across different thresholds.

## 3. Results

### 3.1. Descriptive Statistics

Table 1 presents the sample size by country. We included the data of 1417 cataractous patients, comprising 1001 (70.64%) Hungarian and 416 (29.36%) Kosovan patients.

Cataract surgery and IOL implantation in the right eye were performed in 731 patients and in the left eye in 684 patients (Table 2).

### 3.2. Results for the Hungarian Patients

Regression analysis was used to determine the impact of the refractive correction on the distance visual acuity of the patients before the surgery. Based on the results presented in Table 3, in general, refractive correction using eyeglasses or contact lenses explained the change in the distance visual acuity of 33.2% of the patients (R-squared = 0.332). This regression was statistically significant based on the following: F-statistics = 5.36 and *p* = 0.001.

Based on the coefficient results, the correction in the hyperopic patients had a positive impact on visual acuity (B = 0.01), which was statistically significant (*p* = 0.006). There was also a positive impact on the visual acuity in terms of myopic correction (B = 0.01); however, this was not statistically significant. In the patients with astigmatism, we obtained statistically significant results in cases of myopic astigmatism, where the correction had a high positive impact on visual acuity (B = 0.12) (*p* = 0.017). The impact of the correction on visual acuity was also positive in the cases of hyperopic astigmatism (B = 0.02); however, it was not considered statistically significant.

The preoperative uncorrected distance visual acuity (UDVA preop.), preoperative best-corrected distance visual acuity (BCDVA preop.), 1-month postoperative uncorrected distance visual acuity (UDVA 1 month postop.), the best-corrected visual acuity, and the 1-month postoperative best-corrected distance visual acuity (BCDVA 1 month postop.) in Table 4 show that in the Hungarian patients, the preoperative UDVA was 24.7% or 0.25 in decimal units, while the preoperative BCDVA was 54.4% or 0.5 in decimal units. The visual acuity improved significantly after surgery, with a mean of 77.9% or 0.8 of the UDVA and a mean of 85.2% or 0.85 of the BCDVA. Compared to the uncorrected distance visual acuity and the best-corrected distance visual acuity before cataract surgery, our results indicated that the improvement in visual acuity after surgery was significant and that the refractive outcomes were positive.

To analyze the correlation between the variables, we used Pearson’s correlation (Table 5). According to our findings, there was a negative correlation (r = −0.096), which is statistically significant at the 1% level (*p* = 0.003), between the lens thickness and the distance visual acuity after surgery; therefore, the greater the lens thickness before surgery, the lower the postoperative visual outcome.

**Figure 1:**
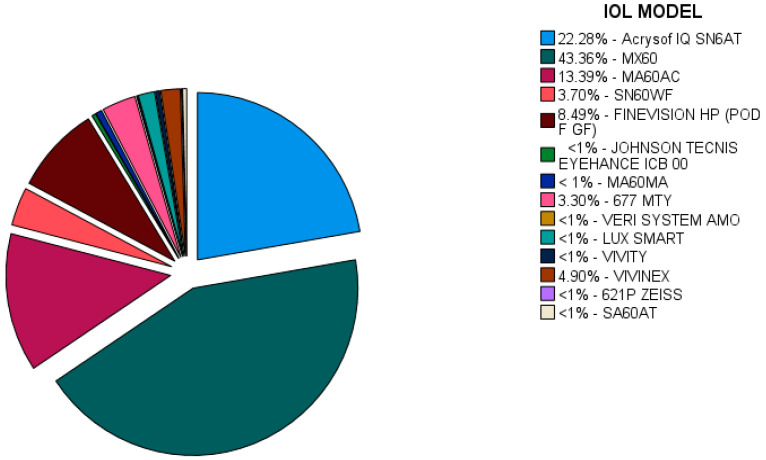
Intraocular lens (IOL) models in Hungarian patients.

To assess the impact of the 14 implanted IOL models (Figure 1) on the postoperative uncorrected visual acuity and best-corrected visual acuity, we used regression analysis. All the subjects were divided into three groups based on the preoperative distance visual acuity, as follows: Group 1—preoperative UDVA = 0–0.33 (0–33%); Group 2—preoperative UDVA = 0.34–0.66 (34–66%); Group 3–preoperative UDVA = 0.67–1.0 (67–100%). We then analyzed the impact of each IOL model on the visual acuity improvement 1 month after the surgery without correction (UDVA 1 month postop.) and 1 month after the surgery with correction (BCDVA 1 month postop.) for each of the three groups.

To evaluate the impact of the IOL model on the postoperative visual outcomes, we divided our patients into three groups based on the preoperative visual acuity: Group 1—0–33% (hand movement—0.33 preoperative visual acuity); Group 2—34–66% (0.34–0.66 preoperative visual acuity); Group 3—67–100% (0.67–1.0 preoperative visual acuity). Our results showed that the AcrySof IQ toric SN6AT (Alcon, Fort Worth, TX, USA) IOL had a statistically significant positive impact on all the groups (*p* < 0.05), with the highest positive impact (B = 0.043) on the 1-month postoperative visual acuity of the second group of patients (0.34–0.6 preoperative visual acuity). However, in all three groups of patients, the AcrySof IQ toric SN6AT IOL had a statistically significant positive impact on the postoperative outcomes (*p* < 0.05); the 1-month uncorrected postoperative visual acuity had a positive coefficient of B = 0.16, while the 1-month best-corrected postoperative visual acuity had a positive coefficient of B = 0.14.

Another IOL model with a significant positive impact on the postoperative visual acuity was found to be the FineVision HP (POD F GF) IOL (BVI, Waltham, MA, USA), similar to the AcrySof IQ toric SN6AT IOL. The highest positive impact was found to be on the second group of patients (B = 0.323). In terms of the total impact on the three groups, the FineVision HP (POD F GF) IOL was found to have a positive impact (B = 0.133) on the uncorrected 1-month postoperative distance visual acuity and an impact of B = 0.0914 on the 1-month best-corrected distance visual acuity, which was also statistically significant (*p* < 0.05).

The IOL used for most of the patients (43.36%), enVista MX-60 (Bausch and Lomb, Bridgewater, NJ, USA), exhibited an overall positive impact on the visual acuity in all the patients. The positive impact on the 1-month uncorrected distance visual acuity was B = 0.0411, whereas the 1-month best-corrected distance visual acuity was B = 0.0245. However, this was not considered statistically significant.

The regression analysis results also indicated the positive impact of the 677MTY (Medicontur Medical Engineering Ltd., Zsámbék, Hungary) IOL on postoperative visual outcomes. In all the groups of patients, the 677MTY IOL had a statistically significant positive impact at the level of 5% (*p* < 0.05), with B = 0.179 for the 1-month uncorrected distance visual acuity and B = 0.139 for the 1-month best-corrected distance visual acuity (Table 6).

### 3.3. Results for Kosovan Patients

Table 7 presents the preoperative distance visual acuity and the 1-month postoperative distance visual acuity of the Kosovan subjects. The mean preoperative distance visual acuity in these patients was 15% or 0.15 in decimal units, whereas the mean uncorrected 1-month postoperative distance visual acuity increased to 44% or 0.4 in decimal units and the best-corrected distance visual acuity increased to 49.6% or 0.5 in decimal units.

In contrast to that observed for the Hungarian patients, there was a positive correlation (r = 0.030) between the lens thickness (LT) and the postoperative visual acuity in the Kosovan patients (Table 8); however, this was not statistically significant.

**Figure 2:**
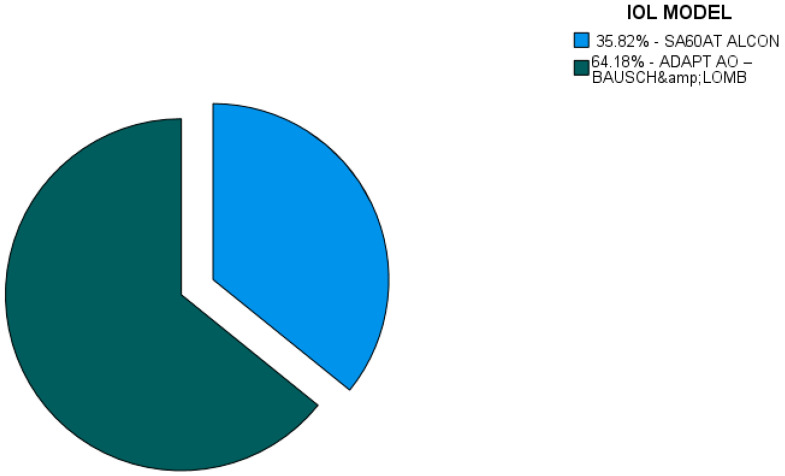
Intraocular lens (IOL) model in Kosovan patients.

The Kosovan patients were treated with one of the two monofocal IOL types, AcrySof SA60AT IOL (Alcon, Fort Worth, TX, USA) and Akreos ADAPT AO (Bausch and Lomb, Bridgewater, NJ, USA), available at the study center. Akreos ADAPT AO was used in 64.18% of the patients and AcrySof SA60AT was used in 35.82% of the cases (Figure 2).

To evaluate the impact of the IOL model on the postoperative visual acuity, we performed a regression analysis. All the patients were divided into three groups based on the preoperative visual acuity. The same division was made as that for the Hungarian patients: Group 1—preoperative UDVA = 0–0.33 (0–33%); Group 2—preoperative UDVA = 0.34–0.66 (34–66%); Group 3—preoperative UDVA = 0.67–1.0 (67–100%). Visual acuity was measured 1 month after surgery in all three groups.

The AcrySof SA60AT IOL was used in 35.82% of the patients and was found to have an overall positive impact on the postoperative distance visual acuity in all three groups. However, there was a higher positive impact on the postoperative distance visual acuity of the patients in the second group, who had a preoperative UDVA from 0.34 to 0.66 (B = 0.321), which was statistically significant.

The other IOL model, Akreos ADAPT AO, was used in 64.18% of the cases, and it was found to have an overall positive impact on the postoperative visual outcomes in all the groups. Similarly to AcrySof SA60AT, Akreos ADAPT AO also resulted in a higher positive impact (B = 0.408) on the patients, whose preoperative distance visual acuity was between 34% and 66%, which was statistically significant at the 5% level. Even though both the IOL models had a statistically significant positive impact on the postoperative distance visual acuity, Akreos ADAPT AO was considered to have a higher positive impact (B = 0.408) than AcrySof SA60AT (Table 9).

### 3.4. Comparison Between Centers

Table 10 shows the results of the statistically significant differences in the LT between the Kosovan and the Hungarian patients using a one-way ANOVA. The mean LT in the Hungarian patients was 4.40 mm, and that in the Kosovan patients was 4.30 mm. The one-way ANOVA revealed statistically significant differences in the mean LT between the Hungarian and Kosovan patients (F-statistics = 10.13 and *p* = 0.001).

Table 11 presents the preoperative UDVA, 1-month postoperative UDVA, and 1-month postoperative BCDVA for all the patients in the two centers. Based on the complete data available from the Hungarian patients, the preoperative UDVA was 24.7% or 0.25, and in the Kosovan patients, the preoperative UDVA was 15% or 0.15. The UDVA improved to 77.92% or 0.8 and the BCDVA improved to 85.21% or 0.85 one month after the cataract surgery and IOL implantation in the Hungarian patients. Improvements in the postoperative visual acuity were also observed in the Kosovan patients: the 1-month postoperative UDVA improved to 43.97% or 0.4, and the 1-month postoperative BCDVA improved to 49.57% or 0.5.

Statistically significant differences between the two groups of patients were found for the 1-month BCDVA. The BCDVA in the Hungarian patients was 85.2%, whereas it was 49.6% in the Kosovan patients.

**Figure 3:**
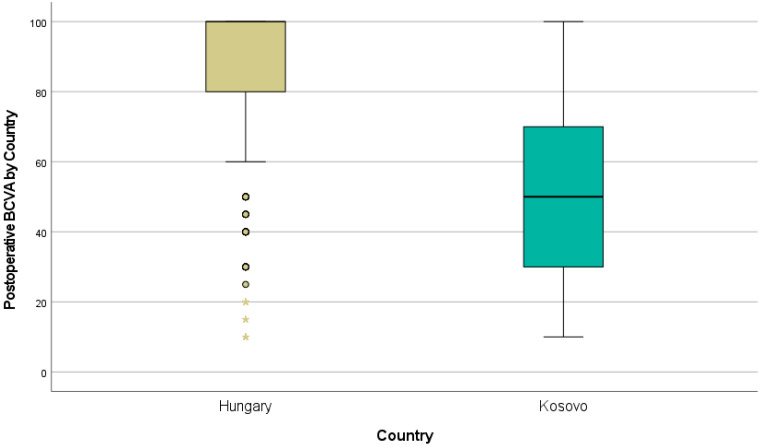
Box plot for statistically significant differences in visual acuity. 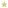 Extreme outliers, which are data points even farther from the rest of the dataset compared to regular outliers. These are values that fall beyond 3 times from the nearest quartile. 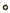 Outliers in the dataset for Hungary. These values deviate notably from the majority of data points because they are much lower compared rest of the data.

Based on the one-way ANOVA results with F-statistics of 568.26 and *p* = 0.001, we can conclude that there is a statistically significant difference in the 1-month postoperative distance visual acuity between the patients from both of the countries (Table 12, Figure 3).

Table 13 presents the results of the impact of the surgeon’s experience on the postoperative outcomes. There was a statistically significant difference in the postoperative results based on the surgeon’s experience. The preoperative UDVA in the Kosovan patients where the surgeon had 11 years of experience in phacoemulsification was 0.15 or 15%, whereas the UDVA in the Hungarian patients before the surgery was 0.25. Based on Table 13, there were statistically significant differences in the 1-month postoperative outcomes between the two groups, according to the particular surgeon. The 1-month postoperative UDVA in the patients treated by the surgeon with 11 years of experience was 0.4 (CI = 0.414–0.467) and the BCDVA = 0.5 or 50% (CI = 0.472–0.521). On the other hand, in the Hungarian patients, the 1-month postoperative outcomes showed a much higher visual acuity, both corrected and uncorrected; the 1-month postoperative UDVA = 0.8 or 80% and the BCDVA = 0.85 or 85%. Based on the *p*-value, where *p* = 0.000, there was a statistically significant difference in the postoperative outcomes based on the surgeon’s experience in phacoemulsification.

## 4. Discussion

The outcomes after cataract surgery are determined by many factors, most of which should be assessed preoperatively. To minimize the MAE owing to the lack of access to the AI-based IOL power calculation formulas for the IOL planning, we included only the IOL calculation data where the SD in the axial length measurement was <0.2 mm. The refractive outcomes after cataract surgery depend on the accuracy of preoperative biometric data, such as AL, K, and ACD; therefore, inaccuracy in their measurement can contribute to 36%, 22%, and 42% of errors, respectively [28]. Another important factor is the accuracy of the IOL power calculation formula; the study of Ferrara et al. found that AI-based formulas and the formulas based on the theory of vergence proved to be more accurate in the IOL power calculation in corneas with low mean keratometry [29].

In our study, we found that the uncorrected postoperative distance visual acuity in the Kosovan patients was only 0.4 or 44% compared to the preoperative visual acuity, which was 0.15 or 15%. Another important aspect of the refractive outcomes was the analysis we conducted of the impact of the IOL model on the postoperative visual acuity of the patients, grouping our patients into three different groups based on their preoperative distance (decimal) visual acuity.

Owing to the unavailability of certain IOL models and premium IOLs, the Kosovan patients were treated with only two types of IOLs: the SA60AT IOL and the ADAPT AO. The superiority of multifocal IOLs has been demonstrated based on the results of many studies [30,31]. Even though both monofocal IOLs had a statistically significant positive impact on the postoperative visual acuity at the 5% level in our Kosovan patients, with ADAPT AO having a higher positive impact (B = 0.408) than SA60AT, the postoperative visual acuity was significantly different between the two groups of patients treated at the two different centers.

Of the 14 IOL models implanted in the Hungarian patients, three had a significant impact on the postoperative distance visual acuity. Our results showed that the AcrySof IQ toric SN6AT IOL (Alcon Laboratories, Inc.) had a statistically significant positive impact on all the groups (*p* < 0.05), with the highest positive impact (B = 0.043) on the second group of patients, who had a 0.34–0.6 preoperative distance visual acuity. The AcrySof IQ toric SN6AT IOL has been widely researched, and many studies found that it has a positive effect on the correction of pre-existing astigmatism [32,33,34]. Scialdone A. et al. [35], in their comparative study of two aspheric toric IOLs, concluded that the patients who were implanted with the AcrySof IQ toric SN6AT IOL were significantly closer to emmetropia postoperatively. Significant positive impact on the postoperative UDVA and BCDVA were also obtained in the cases where FineVision IOL was used. Similarly to the AcrySof IQ toric SN6AT IOL, the highest positive impact was found in the group of patients with 0.34–0.6 preoperative visual acuity (B = 0.323). In terms of the total impact on the three groups, the FineVision HP (POD F GF) IOL resulted in the positive impact of B = 0.133 on the 1-month postoperative distance visual acuity and an impact of B = 0.0914, which was also statistically significant (*p* < 0.05). The FineVision HP (POD F GF) trifocal hydrophobic glistening-free lens also had positive outcomes [36,37,38,39] in other studies.

The enVista MX-60 IOL showed an overall positive impact on the visual acuity in all the patients; the positive impact on the 1-month postoperative distance visual acuity was B = 0.0411, whereas the 1-month postoperative best-corrected distance visual acuity was B = 0.0245. Even though this impact was not statistically significant in our study, in the study conducted by C. Ton Van et al. [40], the incidence of posterior capsular opacification in the cases treated with the enVista MX-60 IOL was only 2.2%, and no glistening was observed. These results confirm the safe profile of this IOL.

The 1-month postoperative distance visual acuity was 0.85 or 85.21% in the Hungarian patients, which was much higher and statistically significant compared to the Kosovan patients. This difference in postoperative outcomes can be attributed to several factors, such as the limited availability of IOL models, the lack of multifocal and toric IOLs, and the economic barriers to timely cataract surgery. Similarly, Zhijian Li et al., in their Heilongjiang Eye Study [41], concluded that the visual outcomes after cataract surgery in northern China were poor and mainly due to economic barriers to cataract surgery uptake. The results of a study from the Swedish National Cataract Register [42] on the factors that might impact postoperative refraction showed that the mean absolute prediction error was related to the study year and that it decreased the more recently the surgery was performed, a result that could be attributed to improvements in the technique and equipment. However, another factor that had an impact on the mean absolute prediction error, according to the results of their study, was preoperative visual acuity. These results correspond to our results on the postoperative visual acuity of Kosovan subjects (44%) compared to Hungarian subjects (85.2%). The lower visual acuity in Kosovan patients can also be explained by the shorter experience with the optical biometer LenStar900 and IOL planning, the lack of experience in the application of premium IOLs, and the overall shorter experience of the surgeon. J. M. Sparrow et al. [43] reported on the importance of both the preoperative factors associated with intraoperative complications and also the importance of having skillful and highly experienced surgeons to manage these cases. In addition, the Auckland Cataract Study [44] confirmed that the risk of intraoperative complications increases when the preoperative risk scores are higher. Furthermore, the findings reported from different studies [45,46,47] emphasize the relationship between surgical complications and worse postoperative visual outcomes. Similarly to the findings in the literature, our results emphasize the importance of the surgeon’s experience in postoperative outcomes. There was a statistically significant difference in the postoperative outcomes between the Hungarian patients treated by a surgeon with 24 years of experience and the Kosovan patients treated by a surgeon with 11 years of experience. The findings of our study align with the findings reported by Haripriya et al. [48] where the complications in the cases with highly experienced surgeons were lower than those in the cases treated by less experienced surgeons.

## 5. Conclusions

Our findings indicate the importance of the IOL model and the surgeon’s experience in the refractive outcomes following cataract surgery. The postoperative outcomes were more than 35% higher in the Hungarian patients than in the Kosovan ones. Our results suggest that better postoperative outcomes are related to the greater experience of the surgeon; in our study, the Hungarian patients treated by a surgeon with 24 years of experience had much more positive postoperative outcomes compared to the Kosovan patients who were treated by a surgeon with fewer years of experience. In conclusion, the availability of premium IOLs, the surgeon’s experience, and the selection of an appropriate IOL based on the individual patients’ needs play a crucial role in better refractive outcomes after cataract surgery.

## 6. Limitations of the Study

To our best knowledge, this study is the first to compare the patients from Kosovo and Hungary. However, several limitations should be taken into consideration in the interpretation of our findings:

Unequal access to premium IOLs: Kosovan patients had no access to premium IOLs (multifocal and toric) and were therefore treated with only one of the two available monofocal IOLs. On the other hand, the Hungarian patients had access to a wider range of IOL models—14 models in total. This disparity in IOL type could significantly influence the refractive outcomes and patient satisfaction. Therefore, the refractive outcomes were statistically significant between the two groups;

Variation in surgeon experience: The Hungarian surgeon had 24 years of experience, whereas the Kosovan surgeon had 11 years, resulting in statistically significant differences between the two groups;

IOL power calculation formulas: This study utilized non-AI-based IOL power calculation formulas. Therefore, the use of AI-based IOL power calculation formulas in future research is expected to improve the accuracy of IOL power determination and postoperative refractive outcomes.

## Figures and Tables

**Table 1:** Sample size.

Country	Sample Size	
	*n*	%
Hungary	1001	70.64
Kosovo	416	29.36
Total	1417	100

**Table 2:** Operated eye.

Surgery	Hungary		Kosovo		Total
	*n*	%	*n*	%	*n*
Right Eye	513	51.35	218	52.4	731
Left Eye	486	48.65	198	47.6	684
Total	999	100	416	100	1415

**Table 3:** Regression analysis of the impact of refractive correction on visual acuity before surgery.

BCDVA Preop.	Coef	St.Err.	*t*-Value	*p*-Value	[95% Conf]	[Interval]	Sig
Hyperopic correction	0.01	0.005	3.03	0.006	−0.01	0.011	***
Myopic correction	0.01	0.002	0.57	0.571	−0.006	0.003	
Hyperopic astigmatism	0.02	0.02	0.12	0.903	−0.038	0.043	
Myopic astigmatism	0.12	0.012	2.45	0.017	−0.036	0.012	**
Constant	0.562	0.04	14.07	0	0.484	0.641	***
Mean dependent var		0.543	SD dependent var			0.291	
R-squared		0.332	Number of obs			820	
F-test		5.369	Prob > F			0.001	
Akaike crit. (AIC)		310.429	Bayesian crit. (BIC)			333.976	

*** *p* < 0.01, ** *p* < 0.05; BCDVA preop.—preoperative best-corrected distance acuity.

**Table 4:** Preoperative visual acuity and 1-month postoperative visual acuity in Hungarian patients.

Variable	Obs	Mean	Std. Dev.	Min	Max
UDVA preop.	911	0.247	0.22	0	0.9
BCDVA preop.	828	0.544	0.291	0.1	1
UDVA 1 month postop.	936	0.779	0.286	0.1	1
BCDVA 1 month postop.	948	0.852	0.258	0.1	1

UDVA preop.—preoperative uncorrected distance visual acuity; BCDVA preop.—preoperative best-corrected distance acuity; UDVA 1 month postop.—1-month postoperative uncorrected distance visual acuity; BCDVA 1 month postop.—1-month postoperative best-corrected distance visual acuity.

**Table 5:** Lens thickness and 1-month postoperative best-corrected distance visual acuity.

Variables		UDVA Preop.	BCVA Preop.	BCVA 1 m Postop.	Lens Thickness	Lens Type
UDVA preop.	Cor	1	0477 **	0.334 **	0.019	0.084 *
	Sig		0.000	0.000	0.565	0.012
	N	911	763	881	911	911
BCDVA preop.	Cor	0.477 **	1	0.402 **	−0.038	0.162 **
	Sig	0.000		0.000	0.273	0.000
	N	763	828	816	828	828
BCDVA 1 month postop.	Cor	0.334 **	0.402 **	1	−0.096 **	0.033
	Sig	0.000	0.000		0.003	0.308
	N	881	816	948	948	948
Lens thickness	Cor	0.019	−0.038	−0.096 **	1	−0.048
	Sig	0.565	0.273	0.003		0.129
	N	911	828	948	1001	1001
IOL model	Cor	0.084 *	0.162 **	0.033	−0.048	1
	Sig	0.012	0.000	0.308	0.129	
	N	911	828	948	1001	1001

Note: ** The correlation is significant at the 0.01 level (1%). * The correlation is significant at the 0.05 level (5%). BCDVA preop.—preoperative best-corrected distance visual acuity.

**Table 6:** Intraocular lens (IOL) model and postoperative visual acuity.

Lens Type	UDVA 1 m postop.	BCDVA 1 m postop.	UDVA 1 m postop.	BCDVA 1 m postop.	UDVA 1 m postop.	BCDVA 1 m postop.	UDVA 1 m postop.	BCDVA 1 m postop.
	0–33 * (Hand Movement—0.33)		34–66 * (0.34–0.66)		67–100 * (0.67–1.0)		Total	Total
SN6AT	0.034 **	0.032	0.043 **	0.03 **	0.008 ***	0.002 **	0.16 **	0.014 **
	(2.67)	(1.14)	(2.12)	(2.22)	(4.53)	(1.99)	(2.14)	(2.85)
MX60	0.0428	0.0168	0.136	0.0521	0.115	0.0594	0.0411	0.0245
	(1.38)	(0.58)	(1.38)	(0.51)	(1.97)	(1.03)	(1.81)	(1.19)
MA60AC	−0.177 ***	−0.0924 *	−0.177	−0.0255	−0.223 **	−0.08	−0.169 ***	−0.0852 **
	(−4.41)	(−2.44)	(−1.77)	(−0.25)	(−3.24)	(−1.20)	(−5.44)	(−3.04)
SN60WF	−0.0564	−0.0736	0.323	0.233	0.133	0.111	−0.073	−0.0658
	(−0.88)	(−1.24)	(1.62)	(1.1)	(1.4)	(1.18)	(−1.51)	(−1.51)
PODFGF PHYSIOL	0.164 **	0.117 *	0.323 *	0.233	0.175 *	0.121	0.133 ***	0.0914 **
	(3.24)	(2.44)	(2.33)	(1.6)	(2.49)	(1.71)	(3.91)	(2.93)
JOHNSON JOHNSON TECNIS EYEHANCE ICB 00	−0.0184	0.188	0.323	0.233	0.204	0.14	0.145	0.146
	(−0.06)	(0.67)	(1.62)	(1.1)	(1.26)	(0.86)	(1.1)	(1.21)
MA60MA	−0.335 **	−0.345 **					−0.397 ***	−0.388 ***
	(−2.74)	(−2.97)					(−3.69)	(−3.94)
677MTY	0.219 **	0.182 *	0.256	0.217	0.16	0.129	0.179 ***	0.139 **
	(2.83)	(2.47)	(1.97)	(1.59)	(1.85)	(1.5)	(3.62)	(3.09)
VERI SYSTEM AMO	−0.0184							
	(−0.06)							
LUX SMART	0.0482	−0.0454	0.223	0.233	0.144	0.12	0.184 *	0.139 *
	(0.28)	(−0.28)	(1.12)	(1.1)	(1.33)	(1.11)	(2.56)	(2.11)
VIVITY	0.182	0.088					0.12	0.0457
	(1.22)	(0.62)					(0.91)	(0.38)
VIVINEX	−0.0851	−0.0787	−0.527	−0.167	−0.646 **	−0.26	−0.0442	−0.0385
	(−0.84)	(−0.82)	(−1.93)	(−0.78)	(−2.88)	(−1.60)	(−0.69)	(−0.68)
621P ZEISS		−0.312					−0.28	−0.354 *
		(−1.92)					(−1.85)	(−2.56)
SA60AT	−0.218						−0.18	−0.254
	(−1.28)						(−0.69)	(−1.07)
Cons	0.718 ***	0.812 ***	0.677 ***	0.767 ***	0.796 ***	0.860 ***	0.780 ***	0.854 ***
	(28.27)	(34.14)	(9.28)	(10.5)	(17.4)	(19.42)	(42.55)	(51.8)
N	612	619	62	67	118	122	883	895

*** *p* < 0.01, ** *p* < 0.05, * *p* < 0.1. UDVA 1 m postop.—1-month postoperative uncorrected distance visual acuity, BCDVA 1 m postop.—1-month postoperative best-corrected visual acuity, Visual acuity in percentage (%).

**Table 7:** Preoperative and postoperative distance visual acuity.

Variable	Obs	Mean	Std. Dev.	Min	Max
Visual acuity preop.	407	0.15	0.424	0	8
UDVA 1 month postop.	397	0.44	0.216	0.1	1
BCDVA 1 month postop.	397	0.496	0.229	0.1	1

UDVA 1 month postop.—1-month postoperative uncorrected distance visual acuity, BCDVA 1 month postop.—1-month postoperative best-corrected distance visual acuity.

**Table 8:** Lens thickness and postoperative distance visual acuity.

Variables		UDVA Preop.	UDVA 1-Month Postop.	Lens Thickness	Lens Type
Visual acuity preop.	Cor	1	0.608 **	0.076	−0.051
	Sig		0.000	0.126	0.305
	N	407	389	407	407
Visual acuity 1 month postop.	Cor	0.608 **	1	0.030	−0.067
	Sig	0.000		0.556	0.184
	N	389	397	397	397
LT	Cor	0.076	0.030	1	0.076
	Sig	0.126	0.556		0.124
	N	407	397	416	416
Lens type	Cor	−0.051	−0.067	0.076	1
	Sig	0.305	0.184	0.124	
	N	407	397	416	416

Note: ** The correlation is significant at the 0.01 level (1%). UDVA preop.—Preoperative uncorrected distance visual acuity; UDVA 1 month postop.—1-month postoperative uncorrected distance visual acuity.

**Table 9:** Intraocular lens (IOL) model and postoperative distance visual acuity.

Lens Type	UDVA 1 Month Postop.	UDVA 1 Month Postop.	UDVA 1 Month Postop.	UDVA 1 Month Postop.
	0–33 ** (Hand Movement-0.33)	34–66 ** (0.34–0.66)	67–100 ** (0.67–1.0)	Total
AcrySof SA60AT	0.0152	0.321 *	0.101	0.0242
	(1.78)	(1.81)	(0.53)	(1.14)
Akreos ADAPT AO	0.0262	0.408 **	0.11	0.0309
	(1.30)	(2.20)	(0.62)	(1.37)
Cons	0.415 ***	0.575 **	0.743 ***	0.459 ***
	−25.74	−4.74	−5.58	−25.57
N	344	7	16	397

*** *p* < 0.01, ** *p* < 0.05, * *p* < 0.1. UDVA 1 month postop.—1-month postoperative uncorrected visual acuity, Visual acuity in percentage (%).

**Table 10:** Statistically significant differences in lens thickness.

Lens Thickness	Posterior			95% Credible Interval			
	Mode	Mean	Variance	Lower Bound	Upper Bound	F	Sig.
Hungary	4.40	4.400	0.000	4.36	4.43	10.13	0.001
Kosovo	4.30	4.302	0.001	4.25	4.35		

**Table 11:** Preoperative and postoperative visual acuity—Kosovo and Hungary.

Country	Variable	Obs	Mean	Std. Dev.	Min	Max
Hungary	UDVA preop.	911	0.247	0.22	0	0.9
Kosovo		407	0.15	0.424	0	8
Hungary	UDVA 1-month postop.	936	0.7792	0.28673	0.10	1.00
Kosovo		397	0.4397	0.21586	0.10	1.00
Hungary	BCDVA 1-month postop.	948	0.8521	0.25827	0.10	1.00
Kosovo		397	0.4957	0.22920	0.10	1.00

UDVA preop.—Preoperative uncorrected distance visual acuity, UDVA 1 month postop.—1-month postoperative uncorrected distance visual acuity, BCDVA 1 month postop.—1-month postoperative best-corrected distance visual acuity.

**Table 12:** One-way ANOVA for statistically significant differences.

Country	BCVA	Mode	Mean	df	F	Sig.
Hungary	Between Groups	0.852	0.852	2	568.26	0.001
Kosovo	Within Groups	0.496	0.496	1343		

**Table 13:** Statistically significant differences in the postoperative outcomes based on the surgeon’s experience.

UDVA Preop.	Posterior			95% Credible Interval		Significant Differences
	Mode	Mean	Variance	Lower Bound	Upper Bound	
11 Years Experience	0.150	0.150	0.000	0.121	0.180	*p* = 0.000
24 Years Experience	0.247	0.247	0.000	0.227	0.266	
**UDVA 1 month postop.**	**Posterior**			**95% Credible Interval**		**Significant Differences**
	**Mode**	**Mean**	**Variance**	**Lower Bound**	**Upper Bound**	
11 Years Experience	0.440	0.440	0.000	0.414	0.467	*p* = 0.000
24 Years Experience	0.779	0.779	0.000	0.762	0.796	
**BCDVA 1 month postop.**	**Posterior**			**95% Credible Interval**		**Significant Differences**
	**Mode**	**Mean**	**Variance**	**Lower Bound**	**Upper Bound**	
11 Years Experience	0.496	0.496	0.000	0.472	0.521	*p* = 0.000
24 Years Experience	0.852	0.852	0.000	0.836	0.868	

UDVA preop.—Preoperative uncorrected distance visual acuity, UDVA 1 month postop.—1-month postoperative uncorrected distance visual acuity, BCDVA 1 month postop.—1-month postoperative best-corrected distance visual acuity.

## Data Availability

All research data and results are available upon request to the corresponding author, Flaka Shoshi; e-mail: flaka.shoshi@phd.semmelweis.hu.
